# Should Europe have a European infant feeding policy?

**DOI:** 10.1002/jpn3.70493

**Published:** 2026-06-26

**Authors:** Stewart Forsyth

**Affiliations:** ^1^ Formerly, Consultant Paediatrician and Medical Director NHS Tayside Scotland UK; ^2^ Present address: Honorary Professor, University of Dundee Dundee Scotland UK

**Keywords:** compliance, evidence, government, nutrition

It is universally accepted that breastfeeding is important for all newborn infants worldwide; it is also recognised that infant feeding advice should be based on the best scientific evidence and reflect the needs of individual infants and families.^1^, ^2^ Certainty of evidence is a key factor for legitimising health policies and a higher level of certainty will generate greater parental compliance.^3^ When relating breastfeeding benefits to specific clinical conditions it is important that there is a clear understanding between an association with the condition and a direct‐cause‐and‐effect with the condition, with the latter providing the highest certainty of evidence and the former drawing attention to potential evidence that needs further investigation.^4^ With global or national socioeconomic diversity also impacting on clinical outcomes, separating the effect of nutritive and non‐nutritive factors on infant and child health is challenging but necessary. For these reasons it is important that policymaking is viewed as a dynamic process with several moving parts and the key objective is to set policy that reflects the certainty of evidence and the changing needs of the target population.

The approach of the World Health Organisation (WHO) has been to pursue a policy on breastfeeding that is all embracing in terms of global reach, maximum exposure and with a strong element of permanency and this is most evident with their longstanding worldwide recommendation that all infants should be breastfed for 2 years or beyond.^5^ This global recommendation may be viewed as being inclusive and equitable but in the absence of adequate supportive scientific evidence it can be a driver for non‐compliance, especially in medium and high income countries, where clean water, sanitation, food supplies, social support, education and healthcare are more readily accessible.^6^

## 1 INNOCENTI DECLARATION

The ‘2 years or beyond’ breastfeeding policy has its origins in what is referred to as the Innocenti Declaration. This ‘declaration’ took place at a meeting titled ‘Policymakers meeting—Breastfeeding in the 1990s: A global initiative’.^7^ It was held at the Innocenti Centre in Florence on 30 July 1990. The meeting included representation from 30 countries, of which only four were high‐income. Organisational representation included the United Nations Children's Fund (UNICEF), WHO, the United States Agency for International Development (USAID), the Food and Agriculture Organisation (FAO) and the World Bank. The co‐sponsors of the meeting were USAID and the Swedish International Development Authority.

The key output from this meeting, which is now referred to as the Innocenti Declaration on the Protection, Promotion and Support of Breastfeeding, stated that all infants should breastfeed for up to 2 years of age or beyond.^7^ There is an intriguing insight into how this recommendation emerged from one of the Swedish representatives, who revealed that the arguments for the agreed recommendation were more anecdotal than scientific.^8^ Evidence included the perception that children are more harmonious in later life if they are breastfed for 5 years or more. Reference was made to the Qur'an, which states that it is a child's right to be breastfed until the approximate age of 2 years. Finally, a Minister of Health from an African country questioned why his country should have sustained breastfeeding while other countries should not. It was therefore agreed that the overriding concern was for children who are living in the most difficult circumstances, and for a consensus to be achieved it was declared that breastfeeding for 2 years or beyond should be a global recommendation. Subsequently, this Declaration was included in the WHO Global Strategy for Infant and Young Child Feeding, published in 2003 and after a further two decades this remains a key WHO policy recommendation.^5^

However, despite the Innocenti Declaration being celebrated each year during Breastfeeding Awareness Week, most higher income countries are a long way from compliance with this policy and many European countries are not even collecting the data.^9^ In 2022, the American Academy of Pediatrics changed their recommendation for duration of breastfeeding from 1 to 2 years and beyond based on their review of the literature and to be consistent with the WHO recommendation^10^ In 2023, a systematic review that was commissioned by WHO as part of the complementary feeding guideline, reported that there were no significant additional health benefits for the infant or for the mother from breastfeeding beyond 12 months.^11^ This conclusion was also reached by the Scientific Advisory Committee for Nutrition that advises the UK government.^12^ However, despite this confirmatory evidence the WHO Complementary Feeding Guideline reaffirmed the view that breastfeeding for 2 years or beyond is a strong WHO recommendation and this was despite their acknowledgement that the evidence was graded as very low certainty.^11^ This presents a dilemma for governments and for parents on whether they act in accordance with the low certainty WHO assertion of benefit from breastfeeding for 2 years or beyond or choose to focus on breastfeeding from birth to 12 months which is the most critical time for infant exposure to breast milk and the data is universally accepted as being of high certainty.

## 2 POLICY AND EVIDENCE

Medicine is not an exact science^13^ and this is particularly evident in breastfeeding research where randomised controlled trials of breastfeeding are not considered to be ethical and therefore research data is generally obtained through observation studies and systematic reviews. It is also evident that the range of health benefits from breastfeeding may differ across the national income groups because of the diversity of living conditions and this should be incorporated into the policy decision making process. In 2016, *The Lancet* published a paper as part of a Breastfeeding Series which was headlined with the statement that universal scaling up of breastfeeding to recommended levels will save 823,000 lives each year in children before the age of 5 years.^14^ This statement now frequently appears in articles describing the health benefits of breastfeeding, and it is regularly cited by WHO, with two members of staff being co‐authors. However, although the number for lives saved globally was very precise, the study did not provide data on the number of lives saved at regional and national levels which would have enabled greater understanding of how health, social and economic factors may have impacted on lives saved in different geographical settings.

Moreover, for this study to be viewed as relevant to countries with different socioeconomic circumstances several points need to be considered in relation to the method and content of the study. The primary source of infant feeding data was from those countries worldwide with the highest infant and child mortality rates. The original data on the relation of breastfeeding to protection against infectious disease was obtained from what was then a recent meta‐analysis,^15^ however the studies selected for that systematic review were not recent with the median year of publication of the 13 studies being 1994, with the first publication in 1987 and the most recent 2006, and all of the countries were of low income. In the Lancet paper, the statistical analysis did not include any data on complementary feeding or childhood diet during the first 5 years of life, and data on nutritional status of the child including prevalence of malnutrition, wasting and stunting, was not included.^14^ With the absence of key health‐related variables and the dominance of the primary data being from low‐income countries, even with sophisticated statistics, it is difficult to see how this study can inform infant feeding policy in high‐income countries or provide a balanced global perspective to the WHO one‐size‐fits‐all policy of breastfeeding for 2 years or beyond. The problem at present is that current infant feeding literature is not able to answer with reasonable certainty that there are additional health benefits from breastfeeding from 2 years or beyond compared to breastfeeding from birth to 12 months. The answer will require evidence from a direct comparison between these two breastfeeding durations with the data stratified by socioeconomic status and adjusted for other relevant variables.

## 3 EUROPEAN INFANT FEEDING POLICY

As populations evolve policies also need to evolve in order to adapt to the ongoing societal change and this is particularly relevant to infants and young children.^16^ Breastfeeding is a single entity that must be considered within the context of other local nutritive and non‐nutritive factors that may impact on the growth and development of infants and children. European countries do not have the challenges of many other countries worldwide and a European infant feeding policy needs to reflect the current needs of the European population and this should provide a platform for a realistic and compliant policy.

It is government responsibility to ensure that their infant feeding policies are appropriate for their infant and young child population and they should not simply outsource a policy that lacks specific relevance to their health and socioeconomic circumstances. WHO is primarily an advisory organisation, available to assist Governments upon request in strengthening health services^17^ and it does not have sovereign rights at a national level. It is important to respect the rights of children and their parents and it is noted that the principles endorsed by the United Nations Convention on Rights of a Child (UNCRC) are clearly aimed at national governments acknowledging that they are ultimately responsible for the health, support and protection of their young citizens, and this will include the delivery of an effective national and regional infant feeding policy.^18^

Governments are able to consult with their national policymakers, health professionals, food industry representatives and parents and with this degree of local collaboration they can provide clarity of ownership, responsibility and accountability. It is noted that the ‘2 years or beyond’ global breastfeeding policy was originally agreed in a European city but the decisionmakers were predominantly representatives of low‐income countries.^7^, ^8^

The longstanding conflict between WHO and the infant formula industry has been a barrier to the development of consensus between the key stakeholders.^19^ The interface between policymakers, health professionals, industry and parents is critically important and must be inclusive if multi‐professional delivery of effective infant feeding policy is going to be successful. Governance standards need to be visible for both research and policy development and policy recommendations should be optimally balanced to ensure that parents receive advice that is pertinent to their children and their living circumstances.

## 4 CONCLUSION

Making breastfeeding everyone's business across Europe^20^ requires a policy that is appropriate for high‐income countries. Infant feeding remains a contentious issue and achieving consensus may be problematic. With philosophical differences on policy and practice and wide‐ranging interpretation of the data, effective leadership and appropriate consultation is required.^19^ The conflict with the infant formula industry is frequently presented as the principal cause of poor compliance with breastfeeding policy, however, the insensitivity of WHO global policy to individual family needs is a frequent comment at the parental and health professional interface. Policymakers need to appreciate that policies are of little value if they are not compliant with the target population and compliance with parents is dependent upon the evidence being credible and relevant to their life circumstances. Europe is fortunate to be a high‐ income region and from a European perspective infant feeding policy needs to reflect the perceived need of the European population.

## CONFLICT OF INTEREST STATEMENT

Stewart Forsyth has received grants from governments, national research funding organisations, charitable organisations and industry, and has received honoraria and expenses for presenting research findings at conferences in the United Kingdom and abroad. Following his retirement from his clinical post he undertook consultancy work for DSM an international company that manufactures nutrient ingredients, and he specifically advised on fatty acids. Within the last 3 years Stewart Forsyth has received an honorarium from Danone for participation in a discussion on governance and regulation of marketing of infant formulas.
